# Cell Detection Using Extremal Regions in a Semisupervised Learning Framework

**DOI:** 10.1155/2017/4080874

**Published:** 2017-06-14

**Authors:** Nisha Ramesh, Ting Liu, Tolga Tasdizen

**Affiliations:** ^1^Scientific Computing and Imaging Institute, University of Utah, Salt Lake City, UT 84112, USA; ^2^Electrical and Computer Engineering Department, University of Utah, Salt Lake City, UT 84112, USA; ^3^Google, Los Angeles, CA 90291, USA

## Abstract

This paper discusses an algorithm to build a semisupervised learning framework for detecting cells. The cell candidates are represented as extremal regions drawn from a hierarchical image representation. Training a classifier for cell detection using supervised approaches relies on a large amount of training data, which requires a lot of effort and time. We propose a semisupervised approach to reduce this burden. The set of extremal regions is generated using a maximally stable extremal region (MSER) detector. A subset of nonoverlapping regions with high similarity to the cells of interest is selected. Using the tree built from the MSER detector, we develop a novel differentiable unsupervised loss term that enforces the nonoverlapping constraint with the learned function. Our algorithm requires very few examples of cells with simple dot annotations for training. The supervised and unsupervised losses are embedded in a Bayesian framework for probabilistic learning.

## 1. Introduction

Automatic cell detection is a fundamental problem that is useful for numerous cell-based studies and quantifications. Also, cell detection is a preliminary step for solving high-level problems, such as cell segmentation, tracking, and analyzing cell data. Accurately detecting a large number of cells in dense images is challenging, for example, when there is occlusion, cells of interest touch each other. Therefore, it is important to develop robust generic algorithms that can tolerate noise and be used on a variety of cells (different modalities and shapes). Detection based on local image information can be erroneous, since they can be associated with imaging artifacts or noise. Including prior information about the objects to be segmented helps in resolving these issues. The priors, learned from the training data, can then be used to learn strategies to detect cells. The process of annotating complete masks for the cells of interest in the training examples is tedious. Working with minimalistic annotations as in [1], where a dot is placed inside each cell in the training images, may be a more plausible solution. These observations motivate us to study semisupervised methods that use minimal training information.

Semisupervised learning benefits from labeled and unlabeled data during the training. Since generating large labeled datasets is time-consuming, it is profitable to build algorithms that can benefit from unlabeled data. This approach is useful in cases when labeled training data is sparse, but there is a large amount of unlabeled data. Incorporating unlabeled data with sparsely labeled training data can help improve the learning accuracy. The goal of our algorithm is to achieve better results in comparison to those of existing algorithms with a significantly smaller amount of training data in a “semisupervised” framework. We also show that in a transductive setting, when we use all the labeled and unlabeled data, we improve the current state-of-the-art results.

Many algorithms have been proposed for semisupervised learning in general, as well as specific applications [2, 3]. Our work is inspired by the semisupervised approach used for electron microscopy image segmentation based on a merge tree structure [4]. The framework in [4] tries to learn the probability of merging/splitting regions in a hierarchical tree structure, whereas we learn a classifier to predict the probability of a region representing a cell. We extend the work in [4] to solve the detection problem by expressing the nonoverlapping constraint as an unsupervised loss function. The main difference is in our construction of the path loss function, to ensure at most one cell is picked in every path versus monotonically nonincreasing constraints on the merging of regions in [4].

We aim to minimize the need for labeled data by taking advantage of the possible interdependence among the unsupervised data. We transform the intensity images to probability maps, such that the objects can be distinguished from the background. We use ILASTIK [5] to train a pixel classifier to generate probability maps. The MSER detector is used on the probability maps to generate a collection of distinct regions [6]. Every connected component in the image is represented using a hierarchical MSER tree. The MSER tree comprises components over different thresholds, from which a subset of nonoverlapping regions that resemble potential cells is selected as the final segmentation. A dot is placed inside each cell in the training images to contribute to the supervised loss. The major contribution of this paper is formulating the nonoverlapping constraint as a differentiable loss function that can be effectively used to steer the unsupervised search to help find a valid solution. We embed the supervised and unsupervised losses in a Bayesian framework for probabilistic learning. The parameters used in the framework are estimated from the data iteratively. We compare our algorithm extensively with [1], since the authors also use dot annotations as ground truth for training. Most of the current competing methods on cell detection/segmentation use complete cell masks as ground truth for training. Hence, it would not be a fair comparison, because we learn from dot annotations. Similar to [1], we use the MSER region detector to generate candidate cell regions and build a hierarchical representation of the image. One disadvantage in [1] while using reduced labeled data is that it does not directly explore the unsupervised data when searching for the most favorable classification function. Our method exploits the underlying correlation between unsupervised data samples. Also, in [1], the nonoverlapping constraint is embedded in the inference, whereas our supervised loss drives the solution to choose regions similar to those of the cells from the training images, and our novel unsupervised loss enforces that at most only one cell is picked in every path.

We begin with a review of the previous related work on cell detection methods in Section 2. In Section 3, we illustrate our detection framework. Experimental results are shown in Section 4, and we summarize our current work and discuss possible extensions for the future in Section 5.

## 2. Related Work

Detection of cells is a fundamental problem because cell morphology and its characteristics are useful for further studies and analysis. The different types of imaging techniques, stains, cell types, and densities contribute to the variability in the cells (shape, size, and texture) [7]. Since the detection and segmentation problems are often addressed together, we will discuss the significant literature for both.

The most fundamental approach for detecting cell regions is intensity thresholding [8, 9]. For cells with homogeneous intensities, which are in contrast to the background and are distinctly separable, simple global thresholding or adaptive thresholding can be used. In reality, the assumption does not hold true. It is very hard to find cell regions with homogeneous intensities. Thresholding may be used as a preprocessing step for the detection problem. The intensity images can also be transformed to represent some derived features using filtering operations (e.g., filter to detect edges, blob detection filters). Edge detection can be used to generate the boundaries of the cells that represent cell contours [10, 11]. These techniques can be included in the preprocessing steps [12, 13]. Even morphological filtering can be used for preprocessing or postprocessing the images to enable detection of individual cells [14–16].

Region segmentation is a popular approach to detect cells, grouping pixels that belong to a cell. Region-growing algorithms start from selected seed points and augment more connected points to form a labeled image [17]. Region segmentation approaches were initially based on clustering algorithms, where similar image pixels were aggregated in an unsupervised framework. The watershed transform, one of the most popular segmentation approaches, uses edge maps to segment an image into multiple regions/catchment basins [18]. The watershed transform usually results in oversegmentation in the case of cell images. Several algorithms use the watershed transform as an initial step in the segmentation pipeline [19–21]. Belongie et al. [22] used Gaussian mixture models to cluster pixels using color and texture features.

Also, considering candidates from a hierarchy of regions has been demonstrated to be an effective method for cell detection and segmentation [1, 4, 19, 20, 23–25]. Hierarchical image segmentation encompasses image segmentations at different detail levels/thresholds. The segmentations are nested. The higher-level regions can be produced from a combination of regions from segmentations at lower levels. Hierarchical methods preserve the spatial and neighboring information among segmented regions. A number of methods can be used to generate the cell candidates, generally running simple algorithms with different parameters. A graph or tree is constructed in which the edges exist between overlapping regions. The watershed transform, an ultrametric contour map (UCM) [26]; the Felzenszwalb's method [27]; the MSER detector [6]; or any other method can be used to build a hierarchical representation. A conservative global intensity, followed by the watershed transform and a persistence-based clustering (PBC) agglomeration, is used in [28]. In [29], the watershed segmentation with different thresholds gives cell candidates, and a learning algorithm based on conditional random field (CRF) is utilized to find the best ensembles to present the final segmentation results. In [30], UCM is used to get a hierarchical representation; a sparse representation-based cell shape model is learned to encode the high-level shape constraints, which are combined with low-level cellular features to produce probability scores of region segmentation candidates. For cell segmentation from candidates obtained from region merging, the supervised term learns the probability of merging regions using examples of true and false merges [30–33]. Dynamic programming or ILP can be used to find a nonoverlapping set of candidates from a hierarchical representation. In [1], the MSER detector is used to build a hierarchical representation and the costs are learned in a structured SVM framework. The nonoverlapping constraint is enclosed in the inference problem. A novel topological loss function to capture the prior information of the cells is used to learn the cost in the structured SVM framework [23].

Graph-cut methods have also been used to solve the cell segmentation problem. It is efficient to represent an image as a graph, where pixels are the nodes and the edge weights are based on the similarity with neighbors [21, 34]. The segmentation problem is then solved as a graph partitioning problem. To reduce the computational load, the graphical representation has also been constructed using superpixels. Superpixels are a group of pixels in spatial proximity that have similar intensity and texture. Superpixels can be obtained by oversegmentation, thereby preserving the image features [35, 36]. In Zhang et al. [21], a region adjacency graph is constructed using superpixels, and segmentation is inferred by partitioning the graph using correlation clustering.

Cells can also be represented using deformable models. Among such methods, variational image segmentation with level sets has been a prominent choice due to their attractive properties such as adaptive topology, which can naturally evolve. An energy functional is defined based on the intensity and shape information. By minimizing the energy functional, the deformable model evolves. The energy terms need to be chosen carefully to minimize the errors in the solution. An extension of level sets to segment cells is used in [37], but it is computationally expensive. Implicit parametric shape models, namely, disjunctive normal shape models, are used to learn representations of cells accurately, where the time complexity increases with the density of the images [38].

Most recently, fully convolutional networks (FCNs) have been used for cell segmentation. FCNs have been extended for cell segmentation in [39]. The algorithm uses cell masks as ground truth for training the network. In [40], the algorithm learns to predict density maps using FCNs in microscopy images. The FCN is trained using dot annotations in simulated data. Since the dot annotations are each represented by a Gaussian, their method cannot be applied to detect cells with arbitrary sizes and shapes.

This paper proposes a new semisupervised framework for detecting cells. Minimalistic dot annotations are used, a dot being placed inside each cell. Our method is evaluated on four datasets and is able to learn a model that achieves better detection accuracy with limited training data, in our evaluation, despite the variation between the datasets. To show the competence of our method, we compare the segmentation accuracy with that of competing methods that train using complete cell masks for only one dataset, since we do not have a one-to-one match for the data used in the other cases.

## 3. Method

To generate cell candidates, we need to be able to distinguish the cells from the background pixels. We use the cell centers to train a pixel classifier using ILASTIK. The dot annotations are each represented by a Gaussian, where the cell centers represent the peak and its width is determined based on the standard deviation. Using the density map, which is formed by the superposition of all the Gaussians, we pick the positive and negative samples. While selecting the positive samples, we use a small value for the standard deviation to enable us to pick only pixels that are close to the centers of the cells. Similarly, we use a large value for the standard deviation to enable us to pick only the background pixels as negative samples. Due to the limited training size in our experiments, we used only two images to learn the pixel classifier in each dataset. The number of pixels selected depends on the number of cells in the image, which varies with different datasets. A set of candidate regions, **R** = {*R*_1_, *R*_2_,…, *R*_*N*_}, is detected using the MSER detector on the cell probability maps. Let *f* : *R* ^*D*^ → *B*, where *B* = {0, 1} and *D* is the dimensionality of the input vector, be a binary indicator function used to predict regions that represent cells. Based on these predictions, we pick a subset of nonoverlapping regions with high similarity to the class of interest from the training examples. The classifiers are trained in a semisupervised framework. The framework includes supervised quadratic loss that relies on user annotations. Similarly, there is an unsupervised loss that restrains the solution to choose nonoverlapping regions. The details of the algorithm are provided below.

### 3.1. Extremal Region Generation

**Figure 1:**
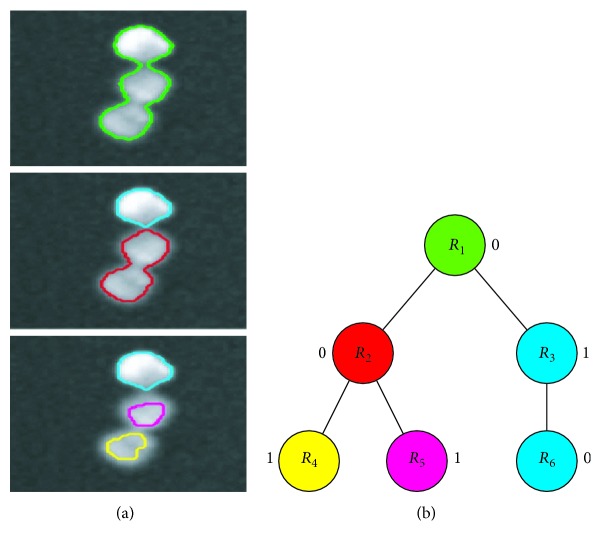
(a) Top-bottom: an example of the boundaries of multiple MSERs that represent the potential cell regions by thresholding the image at increasing values of *t*. (b) The parent-child relationships in the tree correspond to the nestedness of the regions. The tree structure is utilized in our semisupervised framework. The colors indicate individual cell hierarchy.

We use the efficient maximally stable extremal region (MSER) detector on the output of the pixel classifier to find a representative subset of all extremal regions. Extremal regions are regions in which there is a distinct contrast between intensities inside the region and those of its boundary, that is, the average image intensity inside the region is higher or lower than the intensity at the boundary. The image *I* is thresholded at all possible levels. At any level of threshold *t*, the connected components form the set of extremal regions. By having varying thresholds, a pool of extremal regions is generated. In Figure 1(a), we see the image thresholded at increasing values of *t*. An important property of extremal regions is their nestedness. The extremal regions *A* and *B* are nested if *A* ⊂ *B* or *A*⊃*B*. Similarly, regions *A* and *B* are nonoverlapping if *A*∩*B* = ∅. The hierarchical relationships in the tree shown in Figure 1(b) correspond to the nestedness of the regions in Figure 1(a). The tree structure is utilized by our learning framework to determine which of those candidates correspond to cells. The MSER detector from [6] considers only regions that are maximally stable, meaning that stable regions have a lower variation than the regions that are one level below or above. The stability threshold is fixed to a high value to accommodate all potential cell candidates. Similarly, all the parameters of the MSER algorithm are set to enable us to work with the complete tree that can be generated from the image. Hence, regions that have a slight variation from the background are also selected. MSER can extract bright (dark) regions on dark (bright) backgrounds, based on what is selected.

### 3.2. Path Consistency Constraint

The path consistency constraint enforces selection of at most one cell in every path in the tree representation of the connected component. Every image *I* is represented using a forest of trees. Each tree represents a connected region from the image and can contain multiple cells. Let **P** represent all the possible paths in a tree. All paths in a tree do not necessarily have the same depth. For each path *p*_*i*_ ∈ **P** with *n*_*p*_*i*__ nodes, the indices of the regions that comprise the path are given as *d*_*i*_{*d*_*i*_^0^, *d*_*i*_^1^,…, *d*_*i*_^*n*_*p*_*i*_−1_^}. The feature vector for any region *d*_*i*_^*k*^ is represented as **x**_*d*_*i*_^*k*^_. The nonoverlapping loss using a disjunctive normal form to ensure that at most one cell is picked in the *i*th path can be represented as

(1) $$\begin{array}{c} F_{i}=\overset{n_{p_{i}}}{\underset{j=0}{⋁}}\underset{b_{j}}{\underset{⏟}{\overset{n_{p_{i}}-1}{\underset{k=0}{⋀}}f^{j}\left(x_{d_{i}^{k}}\right)}},\\ \;f^{j}\left(x_{d_{i}^{k}}\right)=\left\{\begin{array}{cc} f\left(x_{d_{i}^{k}}\right) & if\;j=k\\ \neg f\left(x_{d_{i}^{k}}\right) & otherwise \end{array}\right., \end{array}$$

where *f*(**x**_*d*_*i*_^*k*^_) represents the score for the region whose indices are given as *d*_*i*_^*k*^. We introduce a binary indicator vector **y** = {*y*_1_, *y*_2_,…, *y*_*N*_} such that *y*_*i*_ = 1 implies the region *R*_*i*_ is picked as a potential cell. An example of a valid combination of regions to represent potential cells for the hierarchical tree in Figure 1(b) is given as **y** = [0 0 1 1 1 0] such that regions *R*_3_, *R*_4_, and *R*_5_ are picked. The path consistency constraint can be easily converted to a differentiable model. We convert the disjunctive normal form into a differentiable model by (1) using DeMorgan's laws to replace unions with intersections and complements; (2) representing the intersections as products; (3) representing the detection classifier using a differentiable classification function, such as an artificial neural network; and (4) relaxing the binary indicator function as $\tilde{f}\left(x_{d_{i}^{k}}\right)$ to estimate the probability of a region to represent a cell. First, the conjunction of binary variables ${⋀}_{k=0}^{n_{p_{i}}-1}\;{\tilde{f}}^{j}\left(x_{d_{i}^{k}}\right)$ is equivalent to the product $\prod_{k=0}^{n_{p_{i}}-1}\;{\tilde{f}}^{j}\left(x_{d_{i}^{k}}\right)$. Next, using DeMorgan's laws, we can express the disjunction ⋁_*j*=0_^*n*_*p*_*i*__^ *b*_*j*_ as negation of conjunctions, ¬⋀_*j*=0_^*n*_*p*_*i*__^ ¬*b*_*j*_, which in turn can be replaced by 1 − ∏_*j*=0_^*n*_*p*_*i*__^(1 − *b*_*j*_). We need to choose $\tilde{f}$ such that it is differentiable. We implement a multilayer perceptron (MLP) with one hidden layer parameterized using **w** as our detection classifier, ${\tilde{f}}_{w}\left(x_{d_{i}^{k}}\right)$. The number of neurons in the input layer is dependent on the size of the data vector (number of features including the bias node described in Section (3.3)). For the hidden layer, we use half the number of nodes used in the input layer. Gradient-based optimization algorithms are used to find the model parameters. The activation function used in the MLP is the tanh function. Finally, the approximate path loss is given as

(2) $$\begin{array}{c} {\tilde{F}}_{i}=1-\overset{n_{p_{i}}}{\underset{j=0}{\prod }}\left(1-\overset{n_{p_{i}}-1}{\underset{k=0}{\prod }}{\tilde{f}}_{w}^{j}\left(x_{d_{i}^{k}}\right)\right),\\ {\tilde{f}}_{w}^{j}\left(x_{d_{i}^{k}}\right)=\left\{\begin{array}{cc} {\tilde{f}}_{w}\left(x_{d_{i}^{k}}\right) & if\;j=k\\ 1-{\tilde{f}}_{w}\left(x_{d_{i}^{k}}\right) & otherwise \end{array}\right.. \end{array}$$

We would like to find **w** so that the predictions, ${\tilde{f}}_{w}\left(x\right)$, satisfy the path consistency constraint, where **x** represents the feature vector of a region.

### 3.3. Bayesian Semisupervised Formulation for Learning

Our framework for learning uses both supervised and unsupervised data. Supervised data corresponds to the regions that represent true cells, generated from the ground truth centroids. Let the supervised data represented by (**X**_s_, **y**_s_) denote the features of the candidate regions and annotations that indicate if the region is a true cell. Unsupervised data refers to the unlabeled images. Since we train with a significantly small amount of labeled data, the unlabeled samples from the training set contribute to the unsupervised data. We can also incorporate more unlabeled data in a “transductive learning” setting by including data from the testing images. Hence, unsupervised data can have contributions from the training images (unlabeled) as well as the testing images (unlabeled). Let **X** be the collection of all supervised and unsupervised samples. The feature vector for every region can be grouped into the following categories:

1. Intensity—histogram of intensities of the region, absolute mean differences, *L*1 and *L*2 distances, and absolute entropy differences between histograms of the region border and a dilation of it for two different dilation radii.
2. Area—areas of the region normalized by the area of the image and perimeters and the boundary length of the regions normalized by the length of the image diagonal.
3. Shape—a shape descriptor using a histogram that represents the variation and distribution of the boundary of the region on a size-normalized polar coordinate system. The curvature and roundness ratio are also computed for the region.
4. Texture—features such as local entropy, standard deviation, and local range of the region are used to describe the texture of the region.

The supervised loss can be represented as an i.i.d. Gaussian *N*(0, *σ*_s_) that penalizes the prediction errors and tries to minimize errors from the true estimate.

(3) $$\begin{array}{c} P\left(y_{s}\;|\;X_{s},w,\sigma_{s}\right)=\frac{1}{{\left(\sqrt{2{\pi \sigma }_{s}^{2}}\right)}^{N_{s}}}\exp\left(-\frac{{\left\|y_{s}-{\tilde{f}}_{w}\left(X_{s}\right)\right\|}_{2}^{2}}{2\sigma_{s}^{2}}\right), \end{array}$$

where *N*_s_ is the number of training samples and *σ*_s_ is the standard deviation that can be estimated while learning the model. The training data helps in avoiding a trivial solution such as the case in which the predictions for all regions are 0. Next, we formulate the unsupervised loss to constrain the solution such that at most one cell may be picked in every path of the MSER tree. For every tree, all possible paths are deduced, and the path function *F* is computed as in (2). Let ${\tilde{F}}_{w}=\left[{\tilde{F}}_{1},{\tilde{F}}_{2},\ldots ,{\tilde{F}}_{N_{p}}\right]$ be the collection of path functions, where *N*_p_ is the number of paths from all the images. The unsupervised loss likelihood is an i.i.d. Gaussian *N*(0, *σ*_u_) that penalizes the difference between each element of ${\tilde{F}}_{w}$ and 1. Let 1 be an *N*_p_ dimensional vector of ones that force the predictions of the classifier to conform to the path constraints.

(4) $$\begin{array}{c} P\left(1\;|\;X,w,\sigma_{u}\right)=\frac{1}{{\left(\sqrt{2{\pi \sigma }_{u}^{2}}\right)}^{N_{p}}}\exp\left(-\frac{{\left\|1-{\tilde{F}}_{w}\left(X\right)\right\|}_{2}^{2}}{2\sigma_{u}^{2}}\right), \end{array}$$

where *σ*_u_ is the standard deviation that can be estimated while learning the model. The standard deviation parameters *σ*_s_ and *σ*_u_ control the contributions of the supervised loss and the unsupervised path loss in the learning framework. They can be tuned using a validation set or estimated from the data. Finally, we include a regularization term to prevent overfitting. This term constrains any abrupt change in the model parameters, thereby establishing the smoothness constraint for the solution.

(5) $$\begin{array}{c} P\left(w\right)=\frac{1}{{\left(\sqrt{2\pi }\right)}^{D}}\exp\left(-\frac{{\left\|w\right\|}_{2}^{2}}{2}\right). \end{array}$$

By applying Bayes' rule, we have the posterior distribution of **w** as

(6) $$\begin{array}{c} P\left(w\;|\;X,X_{s},y_{s},\sigma_{u},\sigma_{s}\right)\propto P\left(w\right)\cdot P\left(1\;|\;X,w,\sigma_{u}\right)\cdot P\left(y_{s}\;|\;X_{s},w,\sigma_{s}\right)P\left(w\;|\;X,X_{s},y_{s},\sigma_{u},\sigma_{s}\right)\propto \frac{1}{{\left(\sqrt{2\pi }\right)}^{D}}\exp\left(-\frac{{\left\|w\right\|}_{2}^{2}}{2}\right)\cdot \frac{1}{{\left(\sqrt{2{\pi \sigma }_{u}^{2}}\right)}^{N_{p}}}\exp\left(-\frac{{\left\|1-{\tilde{F}}_{w}\left(X\right)\right\|}_{2}^{2}}{2\sigma_{u}^{2}}\right)\cdot \frac{1}{{\left(\sqrt{2{\pi \sigma }_{s}^{2}}\right)}^{N_{s}}}\exp\left(-\frac{{\left\|y_{s}-{\tilde{f}}_{w}\left(X_{s}\right)\right\|}_{2}^{2}}{2\sigma_{s}^{2}}\right). \end{array}$$

### 3.4. Estimating the Parameters

We infer the model parameters **w** and *σ*_s_ and *σ*_u_ using maximum a posteriori estimation.

(7) $$\begin{array}{c} w^{*}=\arg\;\underset{w,\sigma_{u},\sigma_{s}}{\max}P\left(w\;\;|\;\;X,X_{s},y_{s},\sigma_{u},\sigma_{s}\right). \end{array}$$

We effectively minimize the negative logarithm of the posterior.

(8) $$\begin{array}{c} L\left(w,\sigma_{s},\sigma_{u}\right)=\underset{E_{w}^{U}}{\underset{⏟}{N_{p}\log\;\sigma_{u}+\frac{1}{2\sigma_{u}^{2}}{\left\|1-{\tilde{F}}_{w}\left(X\right)\right\|}_{2}^{2}}}+\underset{E_{w}^{C}}{\underset{⏟}{\frac{1}{2}{\left\|w\right\|}_{2}^{2}}}+\underset{E_{w}^{S}}{\underset{⏟}{\frac{1}{2\sigma_{s}^{2}}{\left\|y_{s}-{\tilde{f}}_{w}\left(X_{s}\right)\right\|}_{2}^{2}+N_{s}\log\;\sigma_{s}}}, \end{array}$$

(9) $$\begin{array}{c} \left[w,\sigma_{s},\sigma_{u}\right]=\underset{w,\sigma_{s},\sigma_{u}}{\arg\;\min}\left(E_{w}^{U}+E_{w}^{S}+E_{w}^{C}\right). \end{array}$$

The parameters **w** and *σ*_u_ and *σ*_s_ are updated alternatively. Gradient descent is used to update **w**. We alternatively update *σ*_s_ and *σ*_u_ along with **w**. Setting ∂*L*/∂*σ*_u_ = 0 and ∂*L*/∂*σ*_s_ = 0, we can derive closed-form expressions for *σ*_u_ and *σ*_s_.

(10) $$\begin{array}{c} \begin{array}{l} \sigma_{u}=\frac{{\left\|1-{\tilde{F}}_{w}\left(X\right)\right\|}_{2}}{\sqrt{N_{p}}},\\ \sigma_{s}=\frac{{\left\|y_{s}-{\tilde{f}}_{w}\left(X_{s}\right)\right\|}_{2}}{\sqrt{N_{s}}}. \end{array} \end{array}$$

The gradient of (8) with respect to the classifier parameter **w** is

(11) $$\begin{array}{c} \frac{\partial L}{\partial w}=w^{Τ}-\frac{1}{\sigma_{u}^{2}}{\left(1-{\tilde{F}}_{w}\left(X\right)\right)}^{Τ}\frac{\partial {\tilde{F}}_{w}\left(X\right)}{\partial w}-\frac{1}{\sigma_{s}^{2}}{\left(y_{s}-{\tilde{f}}_{w}\left(X_{s}\right)\right)}^{Τ}\frac{\partial {\tilde{f}}_{w}\left(X_{s}\right)}{\partial w},\\ \frac{\partial {\tilde{F}}_{w}\left(X\right)}{\partial w}=\left[{\left(\frac{\partial {\tilde{F}}_{1}}{\partial w}\right)}^{Τ},{\left(\frac{\partial {\tilde{F}}_{2}}{\partial w}\right)}^{Τ},\ldots ,{\left(\frac{\partial {\tilde{F}}_{N_{p}}}{\partial w}\right)}^{Τ}\right],\\ \frac{\partial {\tilde{F}}_{i}}{\partial w}=\frac{\partial }{\partial w}\left[1-\overset{n_{p_{i}}}{\underset{j=0}{\prod }}\left(1-\overset{n_{p_{i}}-1}{\underset{k=0}{\prod }}{\tilde{f}}_{w}^{j}\left(x_{d_{i}^{k}}\right)\right)\right]=-\frac{\partial }{\partial w}\left[\overset{n_{p_{i}}}{\underset{j=0}{\prod }}\left(1-\overset{n_{p_{i}}-1}{\underset{k=0}{\prod }}{\tilde{f}}_{w}^{j}\left(x_{d_{i}^{k}}\right)\right)\right]=-\overset{n_{p_{i}}}{\underset{j=0}{\sum }}\;\underset{r\neq j}{\prod }\left(1-\overset{n_{p_{i}}-1}{\underset{k=0}{\prod }}{\tilde{f}}_{w}^{r}\left(x_{d_{i}^{k}}\right)\right)\frac{\partial }{\partial w}\left(1-\overset{n_{p_{i}}-1}{\underset{k=0}{\prod }}{\tilde{f}}_{w}^{j}\left(x_{d_{i}^{k}}\right)\right)=\overset{n_{p_{i}}}{\underset{j=o}{\sum }}\;\underset{r\neq j}{\prod }\left(1-\overset{n_{p_{i}}-1}{\underset{k=0}{\prod }}{\tilde{f}}_{w}^{r}\left(x_{d_{i}^{k}}\right)\right)\;\overset{n_{p_{i}}-1}{\underset{k=0}{\sum }}\left(\underset{q\neq k}{\prod }{\tilde{f}}_{w}^{j}\left(x_{d_{i}^{q}}\right)\right)\frac{\partial {\tilde{f}}_{w}^{j}\left(x_{d_{i}^{k}}\right)}{\partial w}, \end{array}$$

where ${\tilde{f}}_{w}^{j}\left(x_{d_{i}^{k}}\right)$ represents the detection probability, which is predicted using an ANN as a classifier. After the model parameters are learned, we can use the model, ${\tilde{f}}_{w}$, to predict the detection probabilities. Initially, a random set of weights is chosen, *σ*_s_ = 1. The weights **w** are then updated using gradient descent based only on the supervised loss; *σ*_s_ is also alternatively updated. The supervised loss gives an approximate solution for the weights, which can be fine-tuned using the unsupervised loss. The weights are updated using the gradients computed using the supervised and the unsupervised loss for path consistency, alternatively updating **w** and *σ*_s_ and *σ*_u_. We then select a set of nonoverlapping regions using the inference algorithm described in the next section to identify true cells.

### 3.5. Inference

The probability of a node/region representing a cell *f*_*i*_ is predicted using our classifier. The goal of the inference procedure is to select with minimal cost a subset of nonoverlapping nodes that represent the cells by imposing constraints on the hierarchical tree. Each node in the tree is a potential cell candidate. A label *y*_*i*_ = 1 or *y*_*i*_ = 0 is assigned to each node indicating whether the node is selected as a valid cell. The complete set of labels is represented as **Y**. The detection labels given to all nodes must satisfy the path loss such that at most one cell is picked in every path.

We have explored two approaches to select the cells. Firstly, we used a greedy approach for inferring the final detections. The greedy inference algorithm selects the node with the highest score in a given path predicted using our classifier and assigns it as a label = 1. The other nodes in the path are assigned as a label = 0. The nodes with a label = 1 are the final detected cells.

Alternatively, we can use a bottom-up/top-down algorithm to find the optimal solution imposing the path constraints in the tree structure. If a node *i* is selected, then, *y*_*i*_ = 1 and all of its descendents should be labeled as 0. If the node *i* is not selected, then, problem reduces to finding the best possible cells from the subtrees of the node *i*. All nodes with a label = 1 will be selected as the detected cells. We formulate our detection problem as a constrained optimization problem

(12) $$\begin{array}{c} \underset{Y}{\min}\underset{y_{i}\in Y}{\sum }-y_{i}\;\log\left(f_{i}\right)-\left(1-y_{i}\right)\;\log\left(1-f_{i}\right) \end{array}$$

s.t. if *y*_*i*_ = 1, *y*_*i*′_ = 0, and *i*′ ∈ *D*_*i*_, where *D*_*i*_ is the set of descendent nodes *i*. The optimization problem is solved in a similar manner as seen in [20]. Using the tree structure, we use dynamic programming to find the best and most efficient solution with the path consistency loss. In our bottom-up/top-down algorithm, for inference in the bottom-up step, the minimum energies for both possibilities (region selected/not selected) consistent with the path loss are propagated from the leaves to the root. Then, we choose valid detections by parsing through all the nodes from the root to the leaves.

In the bottom-up step, for every node *i*, a pair of energy sums is computed. The selected energy *E*_*i*_^*s*^ for the *i*th node represents the cost of selecting the node *i* by setting its label = 1 and the labels for all its descendents to 0. Similarly, the not selected energy *E*_*i*_^*ns*^ for the *i*th node computes the cost of not selecting the node *i* by setting its label = 0 and labeling its children optimally subject to the path loss. Let *j* and *k* be the children of the *i*th node. The energies are computed bottom-up in a recursive manner as

(13) $$\begin{array}{c} E_{i}^{s}=-\log\;f_{i}+\underset{k\in D_{i}}{\sum }-\log\left(1-f_{k}\right),\\ E_{i}^{ns}=-\log\left(1-f_{i}\right)+\min\left(E_{k}^{s},E_{k}^{ns}\right)+\min\left(E_{j}^{s},E_{j}^{ns}\right), \end{array}$$

where *D*_*i*_ is a set of indices of descendents of the *i*th node. For leaf nodes, we assign *E*_*i*_^*s*^ = −log(*f*_*i*_) and *E*_*i*_^*ns*^ = −log(1 − *f*_*i*_).  Algorithm 1 gives the pseudocode of the bottom-up algorithm.

After computing the energies for all nodes, we label the regions in a top-down manner. We start from the root node and compare the selected energy *E*_*i*_^*s*^ and the not selected energy *E*_*i*_^*ns*^. If the selected energy is lower than the not selected, then, we select the node *i* by setting its label = 1 and the label for all its descendents to 0. Otherwise, we assign a label = 0 to the *i*th node and search its subtrees.  Algorithm 2 gives the pseudocode of the top-down algorithm. Finally, the nodes with a label = 1 are seen as true detections to give an optimal solution.

We demonstrate the results of using the greedy approach versus the bottom-up/top-down algorithm for inference with our trained classifier for one of the datasets used in our experiments in Table 1. The choice of the methodology of inference does not contribute much to the overall detection scores. Hence, we can choose either of the schemes. We choose the bottom-up/top-down algorithm for our inference.

## 4. Experiments

We evaluate the performance of our detection framework using minimal training data under various conditions to show the individual contribution of the different parts of the algorithm. We also compare the performance of our method in detail to one of the methods that use dot annotations for training their detection classifier.

### 4.1. Datasets

We experiment with four datasets for cell detection. The datasets encompass a wide variety of cells with different shape and intensity characteristics:

1. Phase-contrast images of cervical cancer hela cells from [1]. The dataset contains 11 testing images and 11 training images of size 400 × 400.
2. Synthetic cells from [41]. The dataset contains 20 images. We used 10 images for testing and 10 images for training. The images are of size 950 × 950.
3. *Drosophila melanogaster* Kc167 cells from [41]. The dataset contains 14 images. We used 9 images for testing and 5 images for training. The images are of size 950 × 950.
4. Bright-field images of fission yeast cells from [23, 42]. We did not have access to their complete data. We could obtain only 9 images of size 1024 × 1024. We used only 2 images for training and 7 images for testing.

### 4.2. Implementation Details

For our detection experiments, we do not use the entire labeled training data to learn a model. The probability maps for the images are learned using ILASTIK trained with dot annotations from only two images. The number of cells in each image varies with different datasets. We work with subimages from the training dataset to reduce the manual annotations needed. For every dataset, we create a pool of subimages from the training dataset that contains few cells with their centers annotated. We randomly choose subimages from this pool to train our classifier. The first step is to adapt the training dataset to our algorithm. Since we have only the centroid annotations for the cells, we need to generate ground truth information for every region generated by the MSER detector, which states if the region is a true cell. This can be done by checking all possible regions in a path and selecting the largest regions with a single annotation. The true labels (**X**_s_, **y**_s_) now can be used for the supervised learning in our framework. We have tested the performance of our algorithm when only the training dataset (labeled and unlabeled) is used as well as when we include the testing dataset (unlabeled) along with the training dataset (labeled and unlabeled) to formulate the unsupervised path loss. The weights **w** we learned using the supervised and unsupervised loss are used to evaluate the testing dataset. The candidate regions and their features extracted from the testing images are scored using the learned weights. The label assignment process for the regions in the testing images is based on the inference procedure described in Section 3.5. It takes approximately 48.76 seconds for supervised and 96.59 seconds for semisupervised training for the phase-contrast hela dataset with 10 training images and 10 testing images in MATLAB using 3.5 GHz Intel CPU. We also evaluate the DICE and detection scores for the phase-contrast hela cells when the entire training dataset is used to contribute to the supervised loss. We report results for both cases when we include and exclude the testing dataset in the unsupervised path loss term. We compare our detection and DICE scores with those of other methods.

### 4.3. Evaluation Metrics

We evaluate the performance of our algorithm by computing the detection scores. We find corresponding matching pairs from the pool of ground truth regions and predicted regions using overlapping areas. Similar to [1], we use a Hungarian matching algorithm to find the corresponding areas, by minimizing the distance between their centroids. The oversegmented regions or regions with no correspondences with the ground truth segmentations are labeled as false positives (FP). The unmatched ground truth regions/missed detections are accounted for as false negatives. We evaluate the performance of our algorithm using precision, recall, and *F*-score. Since the ground truth configurations for the regions are generated from the centroids, we do not have true segmentation masks for the cells of all datasets. Hence, we compute the segmentation accuracy using the DICE coefficient for only one of the datasets, for which we had masks.

### 4.4. Results

**Figure 2:**
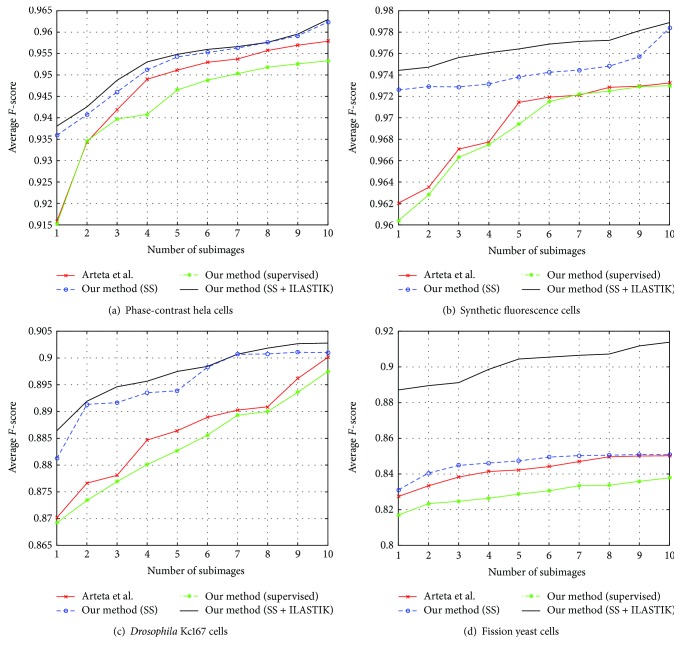
Average *F*-score for varying size of training subimages for our method with only supervised loss (supervised), our semisupervised framework (SS), and our semisupervised framework using ILASTIK as the pixel classifier (SS + ILASTIK) is shown for all the testing datasets. The unlabeled data has contributions from both the training and testing images. We compare the performance of our method with that of Arteta et al. [1].

We compare our algorithm to [1] since the authors also use centroids of cells as user annotations. From all the training images, we generate subimages that are approximately 1/8th the size of the original image. We randomly pick a subset of subimages generated from the training pool and use it to train our supervised loss. Firstly, we evaluate our algorithm using only our supervised loss (our method (supervised)). Next, we train the classifier in our semisupervised framework (our method (SS)). In our semisupervised framework (our method (SS)), we generate the cell candidates using the MSER detector on intensity images. The MSER works well as a region detector only when there is high contrast between the pixels that are inside the cell and those of the background. In cases where the contrast is low, we cannot use the MSER detector on intensity images directly. We can use it on a transformed map of the intensity image in which the contrast is better. Hence, we use the MSER detector on the output of the pixel classifier (ILASTIK). This benefits low-contrast images such as in dataset 4. Finally, we evaluate our algorithm when we generate the cell candidates using the MSER detector on the pixel probability maps trained using ILASTIK (our method (SS + ILASTIK)). We repeat each experiment 20 times to account for the random selection of subimages for the supervised data. Our experiment using ILASTIK (SS + ILASTIK) has been performed in two scenario: firstly, when the contributions to the unlabeled data are only from the training images and secondly, when both the training and testing datasets contribute to the unlabeled data. We report the mean and standard deviation of the *F*-score, mean precision, and recall on the four datasets for both cases in Table 2. We observe that with significantly fewer supervised samples, the classifier benefits from more unlabeled samples. Hence, if we have limited training images, we would benefit by using the unlabeled samples from the testing images. We see that in the case of datasets 3 and 4, we have significantly fewer training images, and hence, using the unlabeled data from the testing dataset improves the performance considerably. Alternatively, in datasets 1 and 2, where we have more unlabeled data from the training images, we see a marginal improvement when we use the testing dataset. We plot the variation in the *F*-score for varying numbers of training subimages in Figure 2. We can clearly see that with the addition of the unsupervised loss, our classifier is able to give better detection scores. We have included the results of using only the supervised loss in our framework to highlight the advantage of using the unlabeled samples.

**Figure 3:**
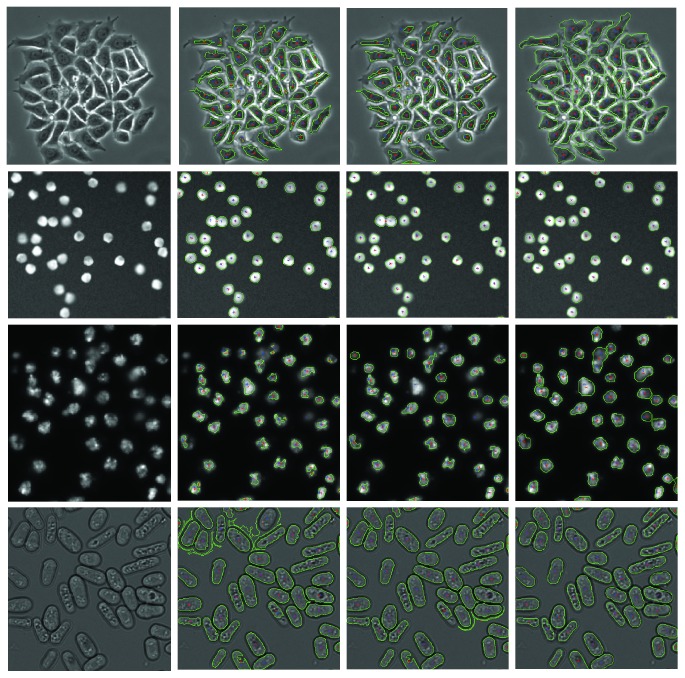
Visual representation of cell boundaries for the different datasets (top-bottom: phase-contrast hela cells, synthetic fluorescence cells, *Drosophila* Kc167 cells, and fission yeast cells). The cell boundaries are shown in green, the predicted center is in red, and the true centroids are in blue. Left-right: input image, results—Arteta et al. [1], results—semisupervised framework (SS), and results—semisupervised framework with pixel classifier (SS + ILASTIK). The unlabeled data has contributions from both the training and testing images.

Our proposed method achieves better accuracy than that of [1] for all four datasets (1, 2, 3, and 4). A visual inspection of our detection with cell boundaries can be seen in Figure 3. We computed the DICE and detection scores for the phase-contrast hela cells using all the labeled images from the training data along with our unsupervised path loss term (Table 3). We observe that our detection scores are comparable to those of the other methods, including [23], which is the state-of-the-art, but we get a better DICE score.

## 5. Conclusion

We proposed a generic semisupervised framework for cell detection that relies on minimal training data. Since our algorithm works with fewer annotations, as the subimages used for training have fewer number of pixels, it reduces the effort required for manually annotating the entire image. The tree framework generated using the MSER detection proves to be very efficient in enforcing our unsupervised loss. The MSER tree framework also limits the search space by considering only regions of desired sizes. By using an ANN as a classifier, we are able to learn complex functions using the supervised and unsupervised loss. We learn the weights for our model by minimizing our cost criterion without the need for parameter tuning. In this paper, we have evaluated our framework only on biological data. Furthermore, we would like to extend this work for time-lapse microscopy images, where we can learn classifiers to predict cell lineages in a semisupervised framework.

## Figures and Tables

**Algorithm 1:**
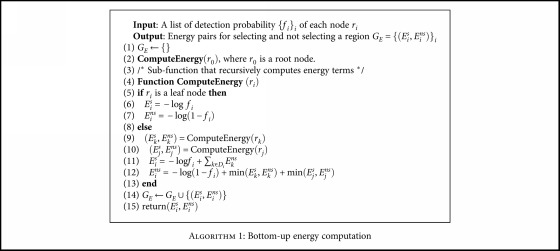
Bottom-up energy computation

**Algorithm 2:**
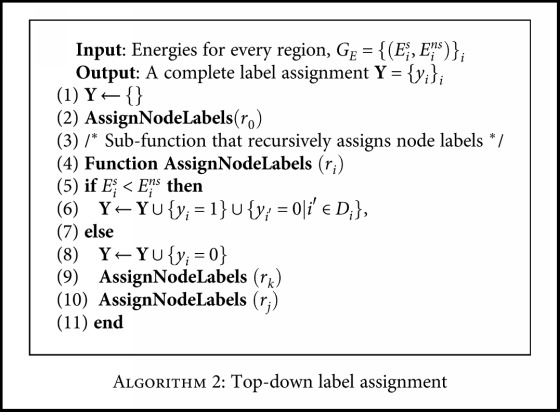
Top-down label assignment

**Table 1:** Comparison of inference methods for phase-contrast hela cells. SS + ILASTIK refers to our semisupervised method using pixel probability maps from ILASTIK. The unlabeled data has contributions from both the training and testing images.

	SS + ILASTIK (greedy inference)			SS + ILASTIK (bottom-up/top-down inference)		
Number of subimages	Precision	Recall	*F*-score	Precision	Recall	*F*-score
1	0.9262	0.9460	0.9359 ± 0.0074	0.9266	0.9498	0.9381 ± 0.0078
3	0.9378	0.9579	0.9477 ± 0.0068	0.9397	0.9580	0.9488 ± 0.0064
5	0.9495	0.9605	0.9549 ± 0.0065	0.9498	0.9599	0.9548 ± 0.0066
7	0.9510	0.9625	0.9567 ± 0.0081	0.9513	0.9620	0.9566 ± 0.0076
9	0.9526	0.9659	0.9592 ± 0.0052	0.9531	0.9661	0.9596 ± 0.0050

**Table:** (a) Phase-contrast hela cells

	Arteta et al. [1]			SS + ILASTIK (training)			SS + ILASTIK (training + testing)		
Number	Prec	Rec	*F*-score	Prec	Rec	*F*-score	Prec	Rec	*F*-score
1	0.9065	0.9253	0.9158 ± 0.0203	0.9180	0.9351	0.9264 ± 0.0082	0.9266	0.9498	0.9381 ± 0.0078
3	0.9342	0.9497	0.9419 ± 0.0084	0.9360	0.9511	0.9434 ± 0.0057	0.9397	0.9580	0.9488 ± 0.0064
5	0.9478	0.9545	0.9511 ± 0.0092	0.9480	0.9568	0.9523 ± 0.0061	0.9498	0.9599	0.9548 ± 0.0066
7	0.9481	0.9594	0.9537 ± 0.0091	0.9500	0.9596	0.9547 ± 0.0091	0.9513	0.9620	0.9566 ± 0.0076
9	0.9536	0.9603	0.9570 ± 0.0055	0.9528	0.9632	0.9579 ± 0.0080	0.9531	0.9661	0.9596 ± 0.0050

**Table:** (b) Synthetic fluorescence cell images

	Arteta et al. [1]			SS + ILASTIK (training)			SS + ILASTIK (training + testing)		
Number	Prec	Rec	*F*-score	Prec	Rec	*F*-score	Prec	Rec	*F*-score
1	0.9695	0.9527	0.9620 ± 0.0106	0.9730	0.9611	0.9670 ± 0.0034	0.9791	0.9698	0.9744 ± 0.0026
3	0.9716	0.9626	0.9671 ± 0.0086	0.9755	0.9662	0.9708 ± 0.0015	0.9801	0.9712	0.9756 ± 0.0005
5	0.9787	0.9643	0.9714 ± 0.0051	0.9794	0.9689	0.9741 ± 0.0013	0.9806	0.9723	0.9764 ± 0.0003
7	0.9792	0.9651	0.9721 ± 0.0036	0.9800	0.9699	0.9749 ± 0.0009	0.9811	0.9732	0.9771 ± 0.0006
9	0.9808	0.9652	0.9730 ± 0.0009	0.9806	0.9701	0.9753 ± 0.0010	0.9813	0.9750	0.9789 ± 0.0004

**Table:** (c) *Drosophila* Kc167 cells

	Arteta et al. [1]			SS + ILASTIK (training)			SS + ILASTIK (training + testing)		
Number	Prec	Rec	*F*-score	Prec	Rec	*F*-score	Prec	Rec	*F*-score
1	0.8195	0.9275	0.8702 ± 0.0156	0.8254	0.9306	0.8748 ± 0.0069	0.8311	0.9496	0.8864 ± 0.0043
3	0.8287	0.9337	0.8781 ± 0.0145	0.8358	0.9416	0.8855 ± 0.0082	0.8402	0.9566	0.8946 ± 0.0056
5	0.8402	0.9380	0.8864 ± 0.0083	0.8433	0.9452	0.8913 ± 0.0093	0.8451	0.9568	0.8975 ± 0.0080
7	0.8427	0.9435	0.8903 ± 0.0055	0.8463	0.9475	0.8940 ± 0.0070	0.8499	0.9580	0.9007 ± 0.0032
9	0.8516	0.9456	0.8962 ± 0.0045	0.8519	0.9497	0.8981 ± 0.0052	0.8526	0.9590	0.9028 ± 0.0038

**Table:** (d) Fission yeast cells

	Arteta et al. [1]			SS + ILASTIK (training)			SS + ILASTIK (training + testing)		
Number	Prec	Rec	*F*-score	Prec	Rec	*F*-score	Prec	Rec	*F*-score
1	0.7492	0.9238	0.8274 ± 0.0136	0.8064	0.9230	0.8607 ± 0.0074	0.8528	0.9243	0.8871 ± 0.0053
3	0.7602	0.9342	0.8383 ± 0.0112	0.8145	0.9276	0.8673 ± 0.0089	0.8602	0.9245	0.8912 ± 0.0050
5	0.7622	0.9412	0.8423 ± 0.0076	0.8226	0.9414	0.8779 ± 0.0076	0.8707	0.9409	0.9044 ± 0.0051
7	0.7645	0.9494	0.8470 ± 0.0039	0.8258	0.9465	0.8820 ± 0.0062	0.8736	0.9420	0.9065 ± 0.0039
9	0.7718	0.9460	0.8501 ± 0.0046	0.8287	0.9460	0.8834 ± 0.0058	0.8791	0.9470	0.9118 ± 0.0047

**Table 3:** Comparison of DICE and detection scores for phase-contrast hela cells. SS refers to our semisupervised approach when the cell candidates are generated using the MSER detector on the intensity images. SS + ILASTIK refers to our semisupervised approach using pixel probability maps from ILASTIK to generate cell candidates using the MSER detector. We evaluate our experiments with and without the contributions of the unlabeled data from the testing dataset.

Method	Detection scores	DICE
Arteta et al. [1]	0.90	0.81
Funke et al. [23]	0.97	0.84
Zhang et al. [21]	0.91	0.80
Our method (SS (only training dataset))	0.94	0.80
Our method (SS (training + testing))	0.95	0.82
Our method (SS + ILASTIK (only training dataset))	0.96	0.86
Our method (SS + ILASTIK (training + testing))	**0.97**	**0.86**
